# Innovations in Cancer Therapy: Endogenous Stimuli-Responsive Liposomes as Advanced Nanocarriers

**DOI:** 10.3390/pharmaceutics17020245

**Published:** 2025-02-13

**Authors:** Jazmín Torres, Johanna Karina Valenzuela Oses, Antonio María Rabasco-Álvarez, María Luisa González-Rodríguez, Mónica Cristina García

**Affiliations:** 1 Departamento de Ciencias Farmacéuticas, Facultad de Ciencias Químicas, Universidad Nacional de Córdoba, Haya de la Torre and Medina Allende, Ciudad Universitaria, Science Building 2, Córdoba X5000HUA, Argentina; jaz.torres.006@unc.edu.ar (J.T.); jkvalenzuelao@gmail.com (J.K.V.O.); 2 Unidad de Investigación y Desarrollo en Tecnología Farmacéutica, UNITEFA, Consejo Nacional de Investigaciones Científicas y Técnicas, CONICET, Córdoba X5000HUA, Argentina; 3 Department of Pharmacy and Pharmaceutical Technology, Faculty of Pharmacy, Universidad de Sevilla, C/Prof. García González 2, 41012 Seville, Spain; amra@us.es

**Keywords:** lipid vesicles, stimuli-sensitive nanocarriers, cancer nanomedicine, drug delivery

## Abstract

Recent advancements in nanotechnology have revolutionized cancer therapy—one of the most pressing global health challenges and a leading cause of death—through the development of liposomes (L), lipid-based nanovesicles known for their biocompatibility and ability to encapsulate both hydrophilic and lipophilic drugs. More recent innovations have led to the creation of stimuli-responsive L that release their payloads in response to specific endogenous or exogenous triggers. Dual- and multi-responsive L, which react to multiple stimuli, offer even greater precision, improving therapeutic outcomes while reducing systemic toxicity. Additionally, these smart L can adjust their physicochemical properties and morphology to enable site-specific targeting and controlled drug release, enhancing treatment efficacy while minimizing adverse effects. This review explores the latest advancements in endogenous stimuli-responsive liposomal nanocarriers, as well as dual- and multi-responsive L that integrate internal and external triggers, with a focus on their design strategies, mechanisms, and applications in cancer therapy.

## 1. Introduction

Cancer represents the second most prominent cause of death in the world. According to the latest global cancer statistics from the GLOBLOCAN project in 2020, there were approximately 19.3 million new cases and 10 million cancer deaths worldwide [1]. The main cancer treatment methods include surgery, radiotherapy, chemotherapy, and immunotherapy. However, these methods present adverse side effects, such as fatigue, hair loss, infections, pain, nausea, mucositis, and vomiting, that can significantly reduce the quality of life of cancer patients [2].

In recent years, a new strategy in cancer therapy, known as targeted drug delivery systems, has been developed to improve cancer therapies. These systems include micelles, dendrimers, liposomes (L), solid nanoparticles, carbon nanotubes, silica nanoparticles, and quantum dots [3]. These nanocarriers help overcome the limitations of conventional cancer therapies, such as low specificity, rapid drug clearance, biodegradation, and poor targeting ability. As a result, nanomedicines enable more precise delivery of anticancer drugs, ensuring controlled and stabilized release at the tumor site [4]. Among them, L are the most successful nanocarriers and have found numerous applications in targeted drug delivery. L were first discovered in the 1960s by Alec Bangham and, since then, they have become the most widely used nanomedicine carriers in targeted drug delivery systems [5].

**Figure 1:**
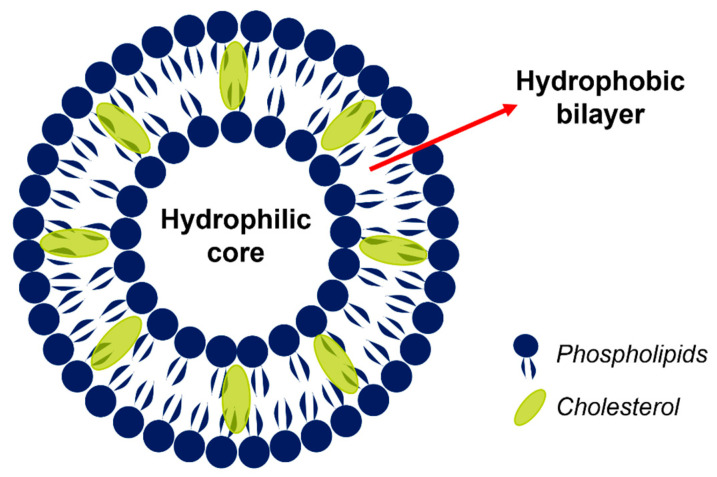
Schematic structure of a liposome. The hydrophilic core is enclosed by a hydrophobic bilayer made of phospholipids and cholesterol, enabling the delivery of both hydrophilic agents within the core and hydrophobic agents within the bilayer.

L are lipidic carriers that can self-assemble in water, composed of one or more amphiphilic phospholipids [6]. The L consist of an aqueous core surrounded by a phospholipid bilayer, and they can be used to deliver both hydrophilic as well as lipophilic drugs [7], as shown in Figure 1.

The L can be categorized depending on size, the number of bilayers, and the composition. Based on the number of bilayers and their thickness, L are classified into large unilamellar vesicles, small unilamellar vesicles, multilamellar vesicles, and multivesicular vesicles [8]. Another classification includes the charge present on the L surface, so they can be classified into cationic, anionic, or neutral types. Depending on size, within the same chemical composition, L can exhibit different physiological behaviors and activities [9].

L-based drug delivery systems are widely used for the delivery of small molecules and nucleic acids [10]. L exhibit many advantages over other delivery systems, the most remarkable being their biocompatibility and safety due to their similarity to biomembranes [11]. They also improve the solubility of lipophilic and amphiphilic drugs, passively target immune system cells, offer sustained release for systemic and local administration, and exhibit improved tissue penetration [12].

To maximize the therapeutic efficacy of traditional nanocarriers, stimuli-responsive nanosystems have been used as an alternative for tumor-specific drug delivery. These nanocarriers may release the drug through various mechanisms, depending on exogenous or endogenous stimuli at the action site. Exogenous stimuli include magnetic fields, light, electric fields, and ultrasound, among others. Stimuli applied remotely enable a controllable and non-invasive drug release, often synergizing with endogenous mechanisms to enhance delivery precision. Innovative approaches, such as light-based photothermal or photodynamic therapy, magnetic fields, and ultrasound, have shown promise in enabling site-specific drug release while minimizing systemic exposure, highlighting their potential as complementary strategies [13].

In contrast, endogenous stimuli are inherently linked to the disease state, triggering drug release at the target site without the need for external intervention. The primary advantage of internal stimuli is their high specificity and responsiveness to the tumor microenvironment, allowing for precise, controlled, and localized drug delivery. This targeted approach not only enhances therapeutic efficacy but also reduces adverse effects by minimizing off-target drug distribution [14]. These stimuli include changes in pH, adenosine triphosphate (ATP) concentration, hydrogen peroxide (H_2_O_2_), redox potential, hypoxia, enzymes, etc. [15]. These stimuli-responsive nanocarriers may enhance the biodistribution of anticancer drugs, increasing their bioavailability and improving their antitumoral efficacy [16].

This review discusses the recent advancements in various types of endogenous stimuli-responsive L, including pH-responsive, enzyme-responsive, redox-responsive, reactive oxygen species (ROS)-responsive, and hypoxia-responsive L. Dual- and multi-responsive systems are highlighted, as they represent some of the most advanced approaches, often integrating multiple endogenous triggers and, in certain cases, combining with exogenous stimuli to achieve enhanced precision and efficacy in cancer therapy. From an integrated perspective, this review not only addresses liposomal design strategies but also evaluates their practical applications in specific cancer treatments. By combining design, mechanisms, and applications, this comprehensive approach offers a relevant contribution to the field, providing insights that have not been thoroughly covered in previous reports.

## 2. Stimuli-Responsive Nanocarriers

Advancements in nanotechnology and its application in the biomedical field have created an opportunity to improve the efficacy of therapeutic agents. Different types of cancer have been successfully treated using nanocarriers [17]. Anticancer therapeutic agents, such as doxorubicin (Dox), cisplatin (Cis), paclitaxel (Ptx), 5-fluorouracil, mitoxantrone, cantharidin, and irinotecan (IR), are among the drugs that have been widely used in nanomedicine [18]. To achieve their outcomes, nanocarriers should deliver the bioactive compounds (e.g., therapeutic or imaging agents) to the tumor tissues or cancer cells, improving the diagnosis and therapeutic efficacy. From the point of view of cancer therapy, side effects should not be present in the ideal nanomedicine. However, numerous barriers are encountered during circulation or within tumors, such as protein corona formation, degradation, burst release or leakage of cargo, and recognition and clearance by the mononuclear phagocyte system (MPS) [15].

Drug delivery strategies in cancer therapy using nanocarriers have enabled the selection of specific nanosystems capable of efficiently transporting therapeutic agents to tumor tissues [19]. Among these, stealth L, commonly PEGylated or incorporating zwitterionic or glycosylated lipids, have been used to enhance the circulation time and reduce immune clearance, thereby improving tumor accumulation. Similarly, trackable L have emerged as a complementary tool, allowing real-time monitoring of L distribution and drug release, although their applications are primarily associated with external stimuli.

In recent years, the use of stimuli-responsive nanocarriers has further improved drug delivery strategies by enabling targeted release of therapeutic agents in response to specific endogenous or exogenous stimuli at the tumor site. In recent years, improved approaches for cancer therapy have included the use of stimuli-responsive nanocarriers, which can achieve targeted release of therapeutic agents when exposed to different endogenous or exogenous stimuli at the tumor site.

**Figure 2:**
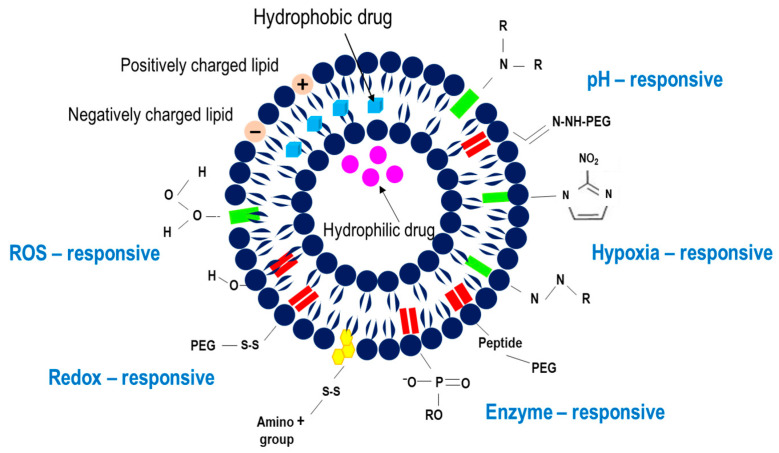
Schematic representation of endogenous stimuli-responsive liposomes.

Systems that respond to endogenous stimuli have been widely used for safe and efficient drug delivery applications in cancer nanomedicine. Based on the promising biomedical applications of these nanocarriers for cancer therapy, this review highlights current and emerging approaches using these stimuli strategies in cancer nanomedicine for delivering bioactive compounds. Special emphasis is placed on endogenous stimuli-responsive L, including pH, enzymes, redox, ROS, and hypoxia (Figure 2). This review also discusses dual- and multi-stimuli-responsive release systems, which provide distinct advantages for drug delivery in cancer nanomedicine, including stealth L. Additionally, some examples of trackable L are discussed as a complementary tool for real-time monitoring of drug distribution and release.

### 2.1. pH-Responsive Liposomes

In the 1980s, researchers defined the concept of pH-sensitive carriers [20]. For the first time, research carried out showed that the tumor microenvironment has a more acidic pH compared to normal tissues. Furthermore, pH-sensitive L can successfully deliver gene fragments and drugs to the cytoplasm through the endocytic pathway [21]. Both are key factors in increasing the drug accumulation on tumors and improving their internalization. Dox is widely used for the treatment of different types of cancers, such as lung, colon, breast cancer, and leukemia, but its use has been limited because it induces cardiomyopathy [22]. pH-sensitive L have been used as a strategy to improve the therapeutic specificity, achieve controlled delivery of drugs, and reduce drug toxicity. L are internalized and taken up by endocytosis, where the cargo ends up fully or partially degraded in the lysosome; thus, endeavors to avoid the process of endocytosis were made [23].

Several researchers have developed pH-sensitive liposomal formulations and studied the mechanism by which L accumulate in the tumor microenvironment and their activity on tumor cells. Table 1 provides a summary of key studies on pH-sensitive L, facilitating a comparative overview. Additionally, some of the most recent and relevant studies are discussed in more detail. For instance, Dos Reis et al. (2021) investigated the viability, internalization, intracellular trafficking, and intracellular delivery mechanism of pH-sensitive Dox-containing L (SpHL-Dox) in human cervical carcinoma (HeLa) cells and reported a gradual decrease in the number of cells reaching 50% of initial values at 24 h. Also, internalization of SpHL-Dox in the presence of pharmacological inhibitors showed that none of the inhibitors reduced the percentage of internalization after 4 h of exposure [24]. Furthermore, studies carried out using acidification inhibitors to evaluate the role of intraluminal pH in the release of Dox from liposomal formulations reported an increased release of Dox when cathepsins were inhibited.

To improve liposomal endocytosis, various ligands can be added to the surface of L based on overexpressed receptors on the surface of cancer cells [60]. A previous study reported that folate receptor-beta (FRβ) is highly expressed in non-small-cell lung cancer on tumor tissues [61]. Furthermore, FRβ is highly expressed in anti-inflammatory M2-polarized tumor-associated macrophages and has been successfully used as a molecular target in therapeutic strategies for lung cancer [62]. In this approach, Park et al. (2021) revealed that docetaxel/doxycycline-loaded FRβ-targeted pH-sensitive L against tumor-associated macrophages and non-small-cell lung cancer showed a synergistic inhibitory effect on tumor growth and suppressing the activity of calpain-2 overexpressed in non-small-cell lung cancer [42].

Similar studies using ligands on the surface of L were reported by Oliveira Silva et al. (2023). They evaluated cytotoxicity, cellular uptake, and in vivo acute toxicity of a novel Dox-loaded folate-coated pH-sensitive L (SpHL-Dox-Fol) in folate receptor (FR+) breast cancer cells (MDA-MB-231 and MCF-7) and FR lung cancer cells (A549). IC_50_ values of 518, 450, and 387 nM were reported for Dox, SpHL-Dox, and SpHL-Dox-Fol, respectively. In addition, SpHL and SpHL-Dox did not show cytotoxicity (~100% of cell viability). Furthermore, cellular uptake was increased when Dox was encapsulated in SpHL-Fol L (30.1 ± 2.7%). Organ toxicity was evaluated in the liver, kidney, and heart. No organ damage was observed with SpHL-Dox-Fol. SpHL-Dox-Fol significantly decreased the systemic toxic effects of Dox [47].

The major drawback of Dox in cancer treatment is its lack of tumor specificity. Nowadays, researchers are trying to modify the existing potent anticancer drugs to improve the specificity of cancer cells, solubility, and efficacy. Specific agents that bind to overexpressed cell surface receptors on the tumor cells have been considered as a strategy to improve Dox selectivity and anticancer activity.

**Figure 3:**
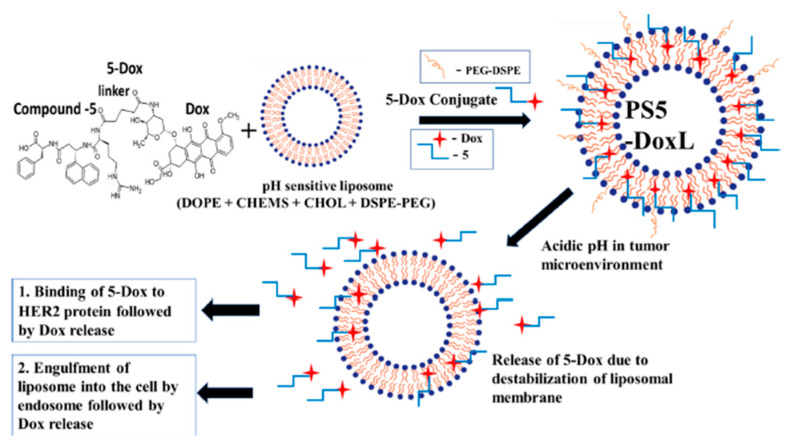
The schematic diagram for preparation of the proposed cancer cell targeting mechanism of a pH-sensitive liposomal formulation containing a peptidomimetic-doxorubicin (Dox) conjugate (PS5-DoxL). DOPE: 1,2-dioleoyl-sn-glycero-3-phosphoethanolamine; CHEMS: cholesteryl hemisuccinate; CHOL: cholesterol; DSPE-PEG: 1,2-Distearoyl-sn-glycero-3-phosphoethanolamine (methoxy(polyethylene glycol)-2000). Reprinted with permission from Ref. [48].

Sonju et al. (2022) constructed a pH-sensitive liposomal formulation containing a peptidomimetic-Dox conjugate (PS5-Dox-L) targeting human epidermal growth factor receptor-2 (HER2)-positive lung and breast cancer cells. These researchers found that release of the drug conjugate and cellular uptake increased in lower pH conditions in HER2-positive cancer cells, as shown in Figure 3 [48]. As mentioned, L are vesicles composed of a lipid bilayer and an aqueous inner, where hydrophilic and hydrophobic moieties are entrapped in the bilayer membrane and aqueous phase, respectively [63]. L differ from each other due to their lipid bilayer composition, which determines their rigidity, size, release rate, and surface charge. For instance, saturated-phospholipid-based L, such as dipalmitoyl phosphatidylcholine, showed high membrane rigidity compared with unsaturated phospholipid-containing lipid membranes [64].

In recent years, the use of L has considerably increased, but there are also some limitations, including mainly their short serum half-life, which has been the biggest challenge that still needs to be surpassed [65]. This limitation can be resolved by conjugating them to polyethylene glycol (PEG) in pH-sensitive L. By combining both strategies, the half-life of the pH-sensitive L in the blood can be increased, along with the release of the drug at the tumor site. Some researchers, such as Alrbyawi et al. (2022), incorporated 1,2-dioleoyl-sn-glycero-3-phosphoethanolamine (DOPE) into pH-sensitive L. DOPE caused the pH-sensitive liposomal formulation to be stable at neutral pH and drug release under acidic conditions due to the destruction of the liposomal bilayer [49].

The daunorubicin-loaded pH-sensitive liposomal system developed was tested in B16 and BL6 melanoma cell lines. Assays of drug release showed a rapid release of daunorubicin from pH-sensitive L at pH 5.5 (50% of the drug was released within 8 h). The pH-sensitive L exhibited a higher cytotoxic and daunorubicin cellular uptake effect on B16 and BL6 cell lines. An alternative to improve efficacy in cancer treatment was to associate Dox with other antitumor drugs; however, this strategy has increased the side effects [66]. Previous studies pointed out that simvastatin (Sim) increases the cytotoxic effect of Dox in breast cancer cell lines [67]. Data reported by Duarte et al. (2021) demonstrated that Sim decreased Dox-induced cardiac toxicity [68]. In this context, the association of both drugs in the liposomal formulation could improve efficacy and reduce toxicity. Recently, Duarte et al. (2023) developed Sim/Dox-loaded pH-sensitive L (Sp-HL-Dox-Sim) and evaluated the effect of different ratios of encapsulated drugs against human breast tumor cell lines, such as MDA-MB 231, MCF-7, or SK-BR-3 cells. Sp-HL-Dox-Sim incubated at pH 5 showed Dox and Sim releases of 90 and 65%, respectively. The increased size of SpHL-Dox-Sim is also indicative that the liposomal formulation responds to pH variation. Co-encapsulation of Dox and Sim at 1:1 and 1:2 in pH-sensitive L, respectively, significantly reduced cytotoxic activity. The IC_50_ value for Sp-HL-Dox-Sim 2:1 was 0.5 in all cell lines investigated. Furthermore, SpHL-Dox-Sim 2:1 showed a synergistic effect in all fractions and against all human breast tumor cell lines evaluated [50].

In addition to their use in drug delivery, pH-sensitive L have emerged as promising platforms for mRNA delivery in cancer immunotherapy. The success of mRNA-based systems, such as SARS-CoV-2 vaccines, has driven the exploration of lipid-based platforms in oncology. In this context, experimental vaccines, like BNT122 (BioNTech) [69] and mRNA-4157 (ModernaTX, Inc., Cambridge, MA, USA) [70], which use lipid nanoparticles (LNPs), have been designed to induce immune responses against tumor-specific antigens in several types of cancer. Although LNPs have shown efficacy in mRNA delivery, pH-sensitive L also represent a versatile alternative, offering specific advantages in terms of stability, controlled release, and compatibility with personalized administration strategies.

The ability of pH-sensitive L to facilitate cytoplasmic release of mRNA in acidic environments makes them key tools in the development of new cancer therapies. For example, Zhang et al. (2020) designed L modified with cholesterol-modified VQWRIRVAVIRK peptide (DP7-C) and 1,2-dioleoyl-3-trimethylammonium propane (DOTAP) to enhance mRNA transfection into dendritic cells, promoting efficient endosomal escape and strong antitumor immune responses in murine lung cancer models [71]. Similarly, Mai et al. (2020) developed L-protamine complexes for intranasal mRNA vaccine delivery, targeting tumor antigens. These systems stabilized and protected mRNA encoding cytokeratin 19, enabling efficient uptake by mucosal dendritic cells and strong activation of CD8+ T-cell responses, resulting in significant tumor growth reduction and metastasis inhibition in preclinical lung cancer models [72].

The convergence of advanced lipid-based technologies underscores their potential as multifunctional platforms for cancer treatment. Initially developed for antiviral applications, like COVID-19 vaccines, these nanoplatforms have been adapted for cancer immunotherapy, highlighting the versatility and transformative impact of pH-sensitive lipid systems in modern biomedicine.

### 2.2. Enzyme-Responsive Liposomes

Enzyme-responsive nanocarriers are nanostructures modified on their surface through the bio-catalytic action of enzymes. This activation improves the internalization of L, leading to accelerated drug release at the target site [73]. Many enzyme-sensitive nanostructures have been used for triggering the delivery of anticancer drugs, including enzyme-responsive L.

Enzyme-responsive L are associated with a selective enzyme located in a target site [74] and they exploit the conformational change of the lipid bilayer associated with these enzymes that, when removed, allow the drug release from L [75].

The enzymes used for enzyme-mediated drug release can be either extracellular or intercellular. Among the many enzymes used to aid drug delivery to cancer, proteases, phospholipases, and glycosidases may be included. Because proteolysis is associated with different diseases, several prodrugs, drug delivery systems, or biomaterials have been designed and developed to exert their activity in endosomal/lysosomal compartments [76]. For instance, cathepsin B is an important element of the lysosome cascade and is involved in tumor invasion and metastasis [77]. One approach that could provide benefits for targeting lysosomes is the application of cationic L. These induce lysosome membrane permeabilization and inhibit efflux. In this context, Lee et al. (2020) synthetized a cathepsin B-responsive L using the GLPG (Gly-Leu-Phe-Gly) oligopeptide for the delivery of Dox in human hepatoma (HepG2) cells. GLPG/Dox-L exhibited low cytotoxicity in HepG2 cells, inhibited cell viability by 60% upon treatment with cathepsin B, and exerted a high anticancer effect in HepG2 cells. In addition, internalization assays of GLPG/Dox-L in HepG2 cells were carried out and the results demonstrated that L were enzymatically degraded by cathepsin B. Consequently, the higher cationic charge on GLPG-L increased the intracellular uptake. Furthermore, anticancer activity of GLPG/Dox-L was confirmed using a zebrafish cancer injection model. Data showed that L caused significant inhibition of cancer cell growth [78].

Phospholipase A2 (PLA2) has also been shown to be overexpressed in a variety of tumors. Researchers have found that secreted PLA2 level is elevated in various inflammatory diseases, atherosclerosis, and cancer [79]. As a result, PLA2-responsive L can be considered promising carriers for the targeted delivery of antitumoral drugs. For example, PLA2 is 22-fold higher in prostate cancer than in normal tissues [80]. On approach, Shchegravina et al. (2019) developed colchicinoid-containing phospholipid prodrugs encapsulated into phosphatidylcholine-based enzyme-responsive L and evaluated their exposure to elevated levels of two kinds of PLA2, porcine pancreas PLA2 and PLA2 from Vipera ursinii venom. A slow rate of hydrolysis of the prodrugs by porcine pancreas PLA2 was revealed. Furthermore, L were tested again in pancreatic cell lines (PANC-1, BxPC-3, and Colo-357), and non-transformed immortalized keratinocytes (HaCaT) showed high cytotoxic activity in the nanomolar concentration range in all tested lines, which implies that L were internalized and processed by cells, leading to drug release. To enable specific delivery of L to target cells, these can be functionalized with ligands capable of selectively binding to receptors overexpressed on the plasma membrane of the tumor [81].

Many ligands, such as antibodies or genetically engineered antibody mimetic proteins, can be used [82]. For example, peptide nucleic acids (PNAs) are artificial nucleic acids with a peptide backbone instead of a sugar phosphate backbone of DNA or RNA that exhibit resistance to degradation, selectivity, and greater binding affinity, in comparison to usual nucleic acids, and can be considered as promising antisense agents in a range of cell models. However, PNAs can be limited by low solubility, poor intracellular delivery, and renal clearance, and cannot efficiently cross the cell membrane without assistance [83]. Cell-penetrating peptides, such as octaarginine, can help to enhance cellular uptake to PNAs. So, Ghavami et al. (2020) studied phospholipase-sensitive L formulation of octa-D-arginine-conjugated antisense PNAs and the effect of PLA2 on drug release and cellular activity. These researchers demonstrated that phospholipase-sensitive L can release PNAs using PLA2 in serum-free conditions. Results showed efficient liposomal encapsulation as well as enzyme-triggered release, resulting in a good cellular antisense effect in HeLa cells [84].

Regarding surface modification with molecules sensitive to enzymes present in the tumor, Wang et al. (2020) constructed an active transporting liposomal nanocarrier using glutathione (GSH)-modified DOPE phospholipid (DOPE-GSH), hydrogenated soy phosphatidylcholine (HSPC) phospholipid, and cholesterol (CHOL) with ɣ-glutamyl transpeptidase (GGT)-triggered transcytosis to encapsulate Dox. The liposomal formulation exhibited potent antitumor activity in mouse tumor models, resulting in complete eradication of hepatocellular carcinoma and cessation of pancreatic ductal adenocarcinoma progression. Impaired hemoperfusion to pancreatic tumors severely restricts drug delivery, and only a low percentage of encapsulated drugs is bioavailable to tumor cells as a result of the slow-release process. Thus, L that exploit the enhanced permeability and retention (EPR) effect, overcome the hypovascular barrier, and release the loaded drugs to target cells would improve drug delivery efficiency [85]. Recently, enzyme-activated prodrug-based nanosystems have been used to improve drug delivery efficiency.

Duan et al. (2021) developed enzyme-activated prodrug-based L to deliver phosphorylated calcipotriol and Dox against pancreatic cancer cells and activated pancreatic stellate cells. These L incorporated a membrane-type 1 matrix metalloproteinase (MT1-MMPs)-cleavable spacer, facilitating tumor-targeted activation. Upon activation by MT1-MMPs expressed in tumor endothelial cells, the L released cyclic peptide targeting integrin αⅤβ3, enhancing tumor angiogenesis and increasing liposomal accumulation and distribution by 3.4-fold. This system achieved a 5.9-fold reduction in tumor weight in preclinical models with minimal side effects, offering a highly efficient and targeted therapeutic approach for pancreatic cancer treatment [86]. Considering that MMPs can degrade the extracellular matrix and most investigated MMPs have expression over 10 to 20 times higher than normal tissues [87], MMPs have been widely investigated. For this reason, they are promising therapeutic targets for cancer therapy [88]. In this line, Juul et al. (2024) designed oxaliplatin-loaded, HER2-targeted, MMP-sensitive L and evaluated the interaction between L and cancer cells by inserting a protease-sensitive cleavable peptide linker into the base of each PEG. These researchers showed that liposomal formulation inhibited tumor growth, improving treatment for HER2-positive cancer [89].

### 2.3. Redox-Responsive Liposomes

It is known that the tumor microenvironment is a highly reducing environment compared to the surrounding healthy cells. In the body, maintaining redox hemostasis is essential for cell survival. This process involves many cellular metabolic pathways, such as glycolysis, GSH synthesis, fatty acid oxidation, and glutaminolysis [90]. In tumor cells, the dysregulated redox hemostasis increased ROS levels, which is associated with high levels of antioxidants. GSH is the main biochemical that causes this reducing nature. GSH levels are over four times greater in tumoral cells than in healthy cells [91]. The elevated level of GSH in different tumors allows the adaptive defense of tumor cells, and the resistance to chemotherapy increases the survival of these cells [92].

**Figure 4:**
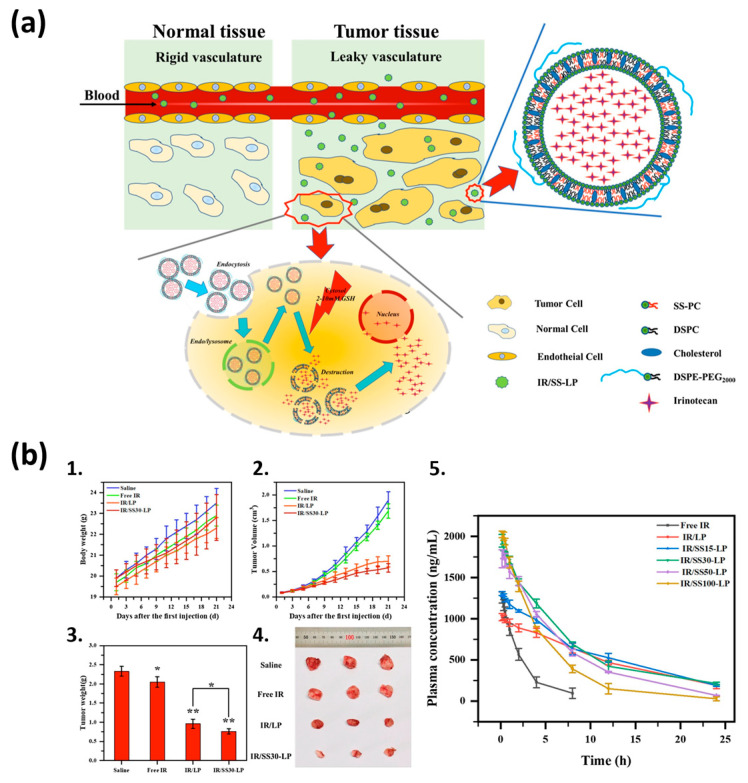
(**a**) In vivo mechanism of redox-responsive irinotecan (IR)-loaded liposomes (L). (**b**) (1–4) In vivo antitumor efficiency test.4T-1 tumor-bearing mice were treated with IR/SS30-LP versus free IR and IR/LP. (1) Tumor volume, (2) body weight, (3) tumor weight, and (4) photographs of tumors (* *p* < 0.05 and ** *p* < 0.01, respectively). (5) The SN38 concentration in plasma after i.v. of free IR and IR/SS-LP L at an IR equivalent dose of 10 mg/kg. SS-PC: disulfide phosphatidylcholine; DSPC: 1,2-distearoyl-sn-glycero-3-phosphocholine; DSPE-PEG2000: 1,2-distearoyl-sn-glycero-3-phosphoethanolamine-N-[methoxy(polyethylene glycol)-2000]; IR/SS-LP: IR/SS-PC liposomes; IR-LP: IR-loaded liposome without SS-PC; IR/SS15-LP: IR/SS-LP with 15% content of SS-PC; IR/SS30-LP: IR/SS-LP with 30% content of SS-PC; IR/SS50-LP: IR/SS-LP with 50% content of SS-PC; IR/SS100-LP: IR/SS-LP with 100% content of SS-PC; SN38: 7-ethyl-10-hydroxycamptothecin. Reprinted with permission from Ref. [94].

Nowadays, scientists have increased their interest in redox-sensitive nanocarriers. These nanocarriers can respond to the high intracellular level of GSH and release the payload in a triggered manner [93]. Drug-loaded nanocarriers include polymeric micelles, polymerosomes, silica nanoparticles, nano-gels, and mainly, L. For example, Wang et al. (2021) developed a novel irinotecan (IR)-loaded redox-responsive L (IR/SS-LP) based on disulfide phosphatidylcholine (SS-PC) for the delivery of IR. This drug was loaded 30% into L, which is a high percentage of loading compared to other antitumor drugs. The redox response of IR/SS-LP revealed that this L formulation released IR in a reduced microenvironment due to the disulfide breakage of disulfide phosphatidylcholine (SS-PC); also, the increased SS-PC content accelerated the drug release of IR/SS-LP. In addition, cellular uptake analysis in MCF-7 cells indicated high internalization efficiency. Additionally, in vitro cytotoxicity assays were conducted using A549 and MCF-7 cell lines in a GSH-supplemented medium. The results showed that IR/SS-LP exhibited significantly higher cytotoxicity against cancer cells compared to non-sensitive L. This highlights that the redox-responsive mechanism, triggered by the elevated GSH levels in tumor cells, efficiently activated drug release, thereby enhancing the cytotoxic effect. Furthermore, in vivo antitumor activity of IR/SS-LP was assessed in 4T-1 tumor-bearing mice, demonstrating superior tumor inhibition compared to free IR and IR/LP (IR-loaded L without SS-PC) at a dose of 5 mg/kg of IR equivalent, with a significantly lower tumor weight in the IR/SS30-LP group [94], as shown in Figure 4.

As described above, the lipid composition of the liposomal membrane is a key factor in controlling drug release. In addition to this, certain trace elements in the human body, such as selenium, have specific biological functions. This compound activates the DNA-damage response to induce the cytotoxicity of tumor cells and increases the apoptosis of cancer cells [95]. To enhance the intracellular drug release, L containing the diselenide (Se-Se) bonds have been developed. Similar to disulfide (S-S) bonds, the Se-Se bonds are cleaved upon exposure to the redox conditions of the tumor microenvironment. However, in comparison to S-S bonds, Se-Se bonds possess a lower bond energy (Se-Se bonds: 172 kJ mol^−1^; S-S bonds: 240 kJ mol^−1^) [96]. Given this, Mirhadi et al. (2022) synthesized Dox-loaded liposomal formulations that contained the redox-sensitive amphiphilic organoselenium compound, 10,10′-diselanediylbis decanoic acid (DDA). In vitro burst release of Dox (more than 30%) in the presence of 0.1% H_2_O_2_ at pH 6.5 was evidenced due to the redox-sensitive role of DDA moieties. The cytotoxicity of redox-sensitive L against colon carcinoma (C26) and fibroblast (NIH-3T3) cell lines was compared with free Dox. All the Dox-loaded L exhibited higher cytotoxicity against C26 cells, with IC_50_ values between 0.03 and 0.06 µg/mL. A high cellular uptake was observed in redox-sensitive L compared to free Dox. Furthermore, the antitumor efficacy study indicated the therapeutic efficacy of all redox-sensitive L compared with free Dox groups (46 vs. 41 days, respectively) [90].

In the last 20 years, the incidence of brain tumors has increased, growing more than 40% in adults [97]. Glioma is the most common malignant primary brain tumor in adults, with a life expectancy of only 14.6 months with comprehensive standard treatment [98]. Traditional treatments, like chemotherapy, are not optimistic due to drugs not crossing the blood–brain barrier (BBB). Lonidamine (LND) was found to have antitumor activity by interfering with energy metabolism, especially its action on tumor mitochondria [99]. The LND is a hexokinase II inhibitor, a key enzyme in the glycolysis pathway. As a chemosensitizer, LND has been used with Dox to improve the efficacy of cancer treatment [100]. In addition, tumors consume large amounts of glucose for their metabolism. Glucose crosses the brain through sodium-independent glucose transporters (GLUTs), which are overexpressed on the surface of tumor cells [101]. Also, cancer cells have more hyperpolarized mitochondrial membrane potential (−220 mV) than normal cells. Consequently, the uptake of lipophilic cationic compounds, such as triphenylphosphonium (TPP), could accumulate in the mitochondria of tumor cells [102].

In recent years, drug delivery systems using receptors and transporters have been shown to provide high drug stability, sustained release potential, low toxicity, and the ability to cross the BBB [103]. Regarding this, Peng et al. (2021) used glucose and TPP to modify multitargeted redox-sensitive L and effectively deliver DOX and chemosensitizer LND for anti-glioma therapy. Data of cellular and mitochondria uptake on (bEnd3) immortalized mouse brain endothelial cells and rat glioma (C6) cells showed that affinity between glucose and brain GLUTs type 1 and positive charges of TPP could enhance the internalization of L. Cytotoxicity and apoptosis studies of L co-modified with glucose and TPP presented an obvious stronger ability to induce C6 cells’ apoptosis and necrosis and lower toxicity on normal cells [104]. Furthermore, modified L have a great mitochondrial targeting ability and synergistic therapeutic effects on glioma. In the past decades, some cell markers have been used in cancer treatment; for example, CD133 and EpCAM have been widely studied as stem cell markers in liver cancer [105].

Some researchers also recognized that peptide ligands are surface-modifying elements used as targeted-delivery therapeutic approaches [106]. Herein, Wang et al. (2020) synthesized a novel CD133/EpCAM-targeted L, capable of co-delivering Dox and salinomycin for the synergistic treatment of liver cancer. Results showed that a high concentration of GSH induced L cleavage and, therefore, release of Dox and salinomycin, inhibiting tumor growth. EpCAM and CD133 peptides allowed for receptor-mediated endocytosis, thereby increasing the L uptake and internalization by cancer stem cells [107]. This suggests that the dual-targeted peptide-mediated cellular uptake and GSH-triggered intracellular reductive cleavage improved the release of the loaded drugs. Wu et al. (2022) carried out studies modifying L with moieties. These researchers developed the cell-surface glycoprotein (CD44) and bone-targeting L delivery system decorated with the redox-cleavable polymer and loaded with cytarabine to treat acute myelogenous leukemia. Alendronate-hyaluronic acid (HA)-S-S bonds-cytarabine-L (ALN-HA-SS-AraC-L) had lower IC_50_ values in comparison with other liposomal systems without S-S bonds (0.60 vs. 0.72 µg/mL). In addition, the ALN-HA-SS-AraC-L group reduced the white blood cell amount in bone marrow and blood smear caused by the acute myelogenous leukemia model. Moreover, these formulations prolonged the survival time when placed into lymphoblasts isolated from a female mouse with acute myeloid leukemia (C1498) tumor-bearing spherical colony isolated from a mouse embryo (C57/BL6; 40% survival over 45 days) [108].

Triggering tumor cell death through apoptosis-mediated pathways is one of the main approaches for cancer therapy, but drug resistance restricts the efficacy of apoptosis [109]. Novel cell death pathways are being developed to potentially overcome these drawbacks. Ferroptosis is a highly iron-dependent programmed cell death, different from cell apoptosis, which occurs through the lethal accumulation of lipid-based ROS when GSH-dependent lipid peroxide repair systems are compromised [110]. The increase of intracellular iron to produce ferroptosis in tumor cells was possible using previously synthesized iron-based nanomaterials. However, iron-mediated hydroxyl radical production is restricted by high GSH levels and insufficient H_2_O_2_ in tumors, which compromises Fenton-reaction-dependent ferroptosis [111]. Fortunately, it has been well established that iron redox coupling (Fe^2+^/Fe^3+^) could initiate lipid peroxidation in the absence of H_2_O_2_ via Fenton-reaction-independent ferroptosis [112]. In tumor cells, Fe^3+^ could react with intracellular GSH, resulting in GSH depletion and reducing Fe^3+^ to Fe^2+^. Based on the above findings, Yang et al. (2021) developed novel unsaturated lipid-rich L, which were loaded with a trisulfide bond-bridged-Dox dimeric prodrug (DSSSD) via the iron method gradient, obtaining an L-DSSSD formulation. Internalization of L-DSSSD gradually increased due to the GSH-triggered drug release in tumor cells. GSH depletion and Dox release were higher due to the increase in the trisulfide–thiol exchange reaction. Therefore, L-DSSSD induced the greatest amount of ROS generation and GSH depletion in tumor cells. The mixture of Dox-mediated apoptosis and L-Fe-induced efficient ferroptosis allowed greater tumor inhibition of L-DSSSD [113].

### 2.4. ROS-Responsive Liposomes

Numerous research efforts to understand the mechanisms and progression of tumor development and the complex biology of cancer have been carried out, but strategies against cancer remain a challenge [114].

Researchers have shown that redox imbalance is an important reason for cancer development, progression, and metastasis in human cells [115]. This disruption in redox balance is attributed to the increase of free radicals, predominantly ROS. The increased ROS level is often linked to DNA damage due mainly to mitochondrial DNA mutations and cellular proliferation [116].

The tumoral microenvironment has a high level of ROS in comparison with normal tissue. Therefore, ROS is a meaningful trigger in the development of the ROS-responsive drug delivery system. Dox has been widely used in chemotherapy owing to its strong antitumor efficiency, but its cardiotoxicity limits its use. Doxil^®^ was the first liposomal drug developed by researchers and approved by the FDA to reduce side effects [117]. However, studies demonstrated that this drug increases the level of ROS in tumor cells, which stabilizes hypoxia-inducible-factor-1α (HIF-1α), a factor leading to tumor tolerance to chemotherapy. To approach this, Xu et al. (2019) encapsulated the HIF-1 inhibitor, acriflavine (ACF), and Dox into L (Dox-ACF@L) and constructed bifunctional L. The chemotherapeutic efficiency of Dox-ACF@L at various concentrations was tested in murine colorectal carcinoma (CT26) cells, and the results revealed improved tumor cell inhibition with the HIF-1 inhibitor. In addition, the presence of ACF in Dox-ACF@L significantly decreased P-glycoprotein levels, in comparison with Dox-ACF@L without ACF. This suggests that the combination of Dox and ACF could suppress the expression of chemotherapy-resistance-related proteins [118].

The introduction of sensitive adjuvants could be a simple method to realize stimuli responsiveness. The adjuvant structure changes under triggers and causes the leakage of encapsulated drugs from the L. In recent years, chemical structures activated by ROS, such as thioether, selenium, thioketal, peroxalate ester, and aminoacrylate, were reported. Among them, thioether has attracted attention due to being the simplest and most powerful, with the greatest application potential. For example, Du et al. (2019) synthesized a novel thioether phosphatidylcholine-based ROS-sensitive L for the controlled release of Dox. In vitro cellular uptake was monitored toward MCF-7 and A549 tumor cells, and the results demonstrated that the liposomal formulation had considerable cellular uptake behavior. In addition, in vitro cytotoxicity indicated that the incorporation of phosphatidylcholine in L membranes did not induce cytotoxicity in the presence of H_2_O_2_. Furthermore, stealth L showed improved efficiency when tested against MCF-7 and A549 tumor cells [119].

The combination of chemotherapeutic agents with monoclonal antibodies against cell death protein 1 (PD-1)/programmed death ligand 1 (PD-L1) attracted attention for cancer treatment, however high production costs, immunogenicity, and poor tumor penetration limit their use [120]. Small-molecule inhibitors that block the PD-1/PD-L1 pathway have been developed to overcome these drawbacks [121]. For example, the small-molecule N-[2-[[2-methoxy-6-[(2-methyl-3-phenylphenyl) methoxy] pyridin-3-yl] methylamino] ethyl] acetamide (BMS-202), a PD1/PD-L1 inhibitor developed by Bristol–Myers Squibb, has a potent inhibitory effect because it induces PD-L1 dimerization [122]. However, the use of BMS-202 is limited due to the lack of its appropriate safe use and an effective delivery system. Ptx is a potent antimitotic chemotherapeutic agent widely used in the treatment of various cancers, such as breast, lung, prostate, and ovarian carcinoma, and it improves the tumor immunosuppressive microenvironment through the upregulation of PD-L1 expression. So, the combination of Ptx and BMS-202 might trigger antitumor immune responses induced by Ptx-mediated immunogenic cell death. In this context, Wang et al. (2022) developed a novel ROS-activated L loading of BMS-202 (a small-molecule PD-1/PD-L1 inhibitor) and PSN (aliphatic carbon chain) into the L, obtaining PSN/BMS-202-L for a ROS-sensitive Ptx release. In vitro cytotoxicity of PSN/BMS-202-L was investigated on murine breast carcinoma 4T1 and HeLa cells. This formulation exerted a high cytotoxicity, probably due to ROS-sensitive Ptx release in the tumor cells, suggesting that the thioether activates these derivatives and increases the therapeutic effect of PSN/BMS-202-L. Furthermore, in vivo antitumor effect studies showed that PSN/BMS-202-L induced high necrosis and apoptosis on tumor cells, probably due to the synergism of PSN and BMS-202 [123].

The studies mentioned above were focused on developing formulations to treat breast and lung cancer due to the higher incidence of these around the world in comparison to ovarian cancer [124]. Despite a low incidence of ovarian cancer, relapsed patients can have metastasis and develop resistance and severe side effects after chemotherapy. In view of this, Tang et al. (2024) investigated a synergistic ROS-responsive L to enhance the targeting effect of ovarian cancer in the tumor microenvironment. A novel HA-modified Ptx and diosgenin (Dio) L (HA-Ptx-Dio-L) was developed and tested on human ovarian cancer cells (ID8). Results showed that cellular uptake by ID8 cells increased with the addition of HA. Also, HA-Ptx-Dio-L revealed a significant inhibitory effect on tumor growth [125].

Despite the use of adjuvant structures as inhibitors to efficiently enhance ROS-responsive L, cancer cells adapt to oxidative stress by activating adaptive antioxidants, such as GSH, to protect cells against endogenous and exogenous oxidative stress. Thus, the design of nanosystems to increase oxidative stress and deplete antioxidant species is necessary to kill cancer cells. The amplification of oxidative stress strategy is used most frequently in cancer treatment [126]. Another strategy applied by some researchers was the development of inorganic or organic nanomaterials to increase the oxidative stress of nanoparticles. Transferrin (Tf) receptor is overexpressed on the surface of cancer cells. Tf safely delivers iron through circulation to cells. Tf-bound iron is incorporated through Tf receptor (TfR) 1-mediated endocytosis [127]. Tf carries Fe^+3^, which is reduced to Fe^+2^ metalloreductases in an acidic environment, and this metal is involved in the generation of ROS. In this context, Tf can act as a pilot for targeting the Tf receptor and as a ferric ion carrier to catalyze dihydroartemisinin (DHA). Furthermore, L-buthionine-sulfoximine (BSO) inhibits the production of GSH, weakening the antioxidant defense system of cancer cells [128]. Given this, integrating Tf, DHA, and BSO could enhance the efficacy of ROS-based therapies. Regarding this, Yu et al. (2020) designed and synthesized Tf-decorated, DHA-, BSO-, and CellROX-loaded liposomal nanoparticles (Tf-DBC NPs) to treat liver cancer, analyzing the generation of ROS in HepG2 cells. Results revealed an increase in the generation of ROS. Also, researchers investigated the essential role of iron ion for the generation of ROS and showed that Fe (II) induced ROS generation when cancer cells were treated with Tf-DBC NPs. In addition, assays demonstrated that Tf-DBC NPs induced cell apoptosis due to the presence of Tf [129].

**Figure 5:**
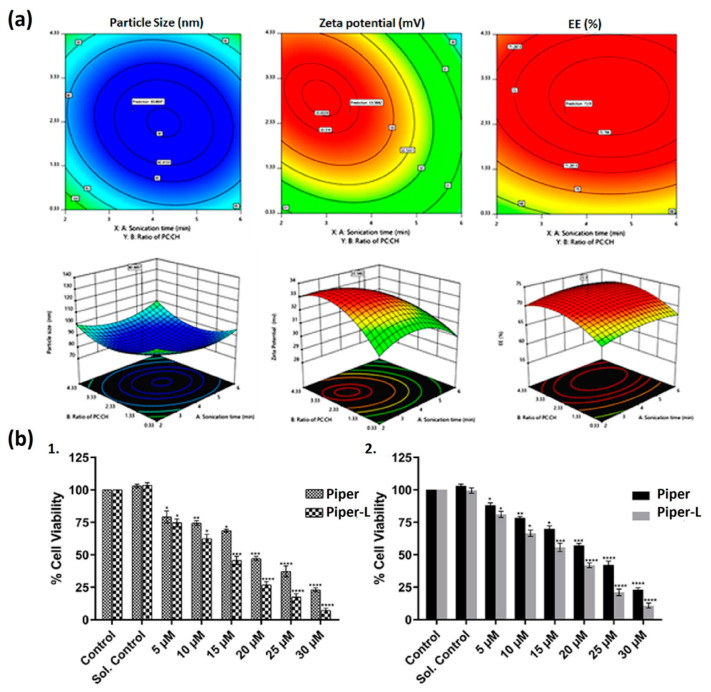
(**a**) Graphical 3D representation of a response surface plot and 2D contour plot demonstrating the effect of sonication time (X1; min), ratio of L-α-phosphatidylcholine (SPC):cholesterol (X2; %), and temperature (X3; °C) on particle size (Y1; nm), entrapment efficiency (EE; Y2; %), and zeta potential (Y3; mV). (**b**) Percent of cell viability after treatment with different concentrations of Piperlongumine (Piper) and Piper-loaded liposomes (Piper-L): (1) SiHa cells and (2) HeLa cells. Data of three independent experiments, shown as mean ± SE (statistical significance: * *p* < 0.05, ** *p* < 0.01, *** *p* < 0.001, and **** *p* < 0.0001). Copyright 2023 Elsevier. Reprinted with permission from Ref. [132].

Almost all previous studies carried out have focused on the encapsulation of synthetic drugs. Nowadays, various recent studies have focused on natural resources of bioactive compounds and tried to recognize their potential against cancer cells [130]. Piperlongumine (Piper) is a well-known alkaloid present in the *Piper longum* plant that possesses biological activity. Piper induces apoptosis by ROS accumulation in cancer cells via different molecular mechanisms. Available studies suggested that Piper induces cytotoxicity in cancer cells mainly through the accumulation of intracellular ROS [131]. In a recent study, Parveen et al. (2023) formulated Piper-loaded L using the thin-film hydration method and optimized them with Design-Expert^®^ through response surface methodology to enhance drug delivery in cervical cancer cells. Using Design-Expert^®^, they optimized variables, such as sonication time, phosphatidylcholine-to-CHOL ratio, and temperature, achieving optimal particle size, encapsulation efficiency, and zeta potential. L demonstrated superior efficacy in inducing ROS production, leading to a redox imbalance that triggered apoptosis in squamous carcinoma (SiHa and HPV-16) and adenocarcinoma (HeLa and HPV-18) cell lines, with apoptosis rates of 63% and 73%, respectively, as shown in Figure 5. Additionally, cell migration assays confirmed that L were more effective in inhibiting cell migration, a critical factor in cancer progression [132].

### 2.5. Hypoxia-Responsive Liposomes

Hypoxia is a pathological characteristic that is observed in 50–60% of solid tumors, in which the tumor cells are deprived of an adequate oxygen supply [133]. The survival of tumor cells is increased by hypoxia through processes such as angiogenesis, invasiveness, and metastasis, and the decreased therapeutic effect and poor prognosis of chemotherapy [134]. Despite this, hypoxic cells can be exploited for therapy by non-toxic, hypoxia-activated prodrugs. Hypoxia-activable prodrugs are activated by the in situ reduction process in hypoxic tumor cells to induce toxicity and have been reported for cancer treatments [135]. However, the therapeutic efficacy of small-molecule drugs is limited in clinical applications. Recently, drug delivery systems based on hypoxia-responsive nanoparticles in response to hypoxic tumor microenvironments for tumor-targeted drug delivery and therapy were developed to solve these problems [136]. For example, Shah et al. (2022) investigated the activity of vinblastine-N-oxide hypoxia-activated prodrug-loaded L (CPD100-L) in pancreatic ductal adenocarcinoma. CPD100-L was tested for their cell proliferative activity under hypoxic conditions (0.1%) in Panc-1 and MiaPaCa-2 pancreatic cells. IC_50_ values of 62 ± 7 nM and 3 ± 0.4 nM at 0.1% oxygen in Panc-1 and MiaPaCa-2, respectively, were shown. They also observed that no effect of empty L on both cell lines existed. To investigate the penetration and apoptotic effect of CPD100-L, 3D spheroids of Panc-1 were used, and results revealed shrinkage and disruption of the spheroids, causing cell death [137]. Some drugs, such as platinum (Pt), are widely applied in chemotherapy treatment. All Pt drugs impair normal DNA functions by generating monoadducts as well as DNA crosslinks [138]. Considering that inhibition of DNA repair by targeted drugs is a pivotal approach to potentiating the therapeutic efficacy of Cis, some researchers, such as Chen et al. (2021), formulated a nanosystem based on glucose oxidase (GOx)/tirapazamine(Tpz)@L-Pt from a platinum (IV) prodrug as a building block, encapsulated payloads of GOBx and the hypoxia activatable dual-function chemo-drug Tpz, and evaluated their efficiency in Cis-resistant tumors. In vitro inhibition demonstrated that GOx/Tpz@L-Pt had a higher oxygen consumption rate within (BEL7404DDP) human hepatocellular carcinoma cells. In addition, therapeutic effects of GOx/Tpz@L-Pt in patient-derived tumor organoids showed that L inhibited the proliferation of tumor organoids, indicating potent efficacy. Furthermore, the in vivo anticancer efficacy of GOx/Tpz@L-Pt in cell-line-derived xenograft models evidenced a potent inhibition of tumor growth. Also, results confirmed that GOx-catalyzed oxygen amplified the intratumor hypoxia. GOx/Tpz@L-Pt led to a complete inhibition of the tumors in mice (82.08%). Furthermore, the liposomal formulation showed markedly reduced sites of liver metastasis (~2 sites per mouse) [139].

Although these prodrugs have greater anticancer efficacy, poor selectivity and rapid elimination are the biggest challenges they are confronted with. Given these points, recent efforts have focused on new compounds, such as nitroaromatics and azo derivatives, due to their sensitivity. Nitroreductase has been widely used in the reduction of nitroaromatic compounds in hypoxic conditions and could be used to activate prodrugs for chemotherapeutic cancer treatment [140]. Herein, Li et al. (2019) studied Dox-loaded nitroimidazole-derivative-incorporated L (Dox-L) for hypoxia-triggered drug delivery. Under hypoxic conditions, nitroimidazole derivative breaks down the phospholipid bilayer, releasing the encapsulated drug, so Dox-L released over 83% of the drug after 12 h. Cytotoxicity-assessed human pharyngeal squamous carcinoma (FaDu) and mouse prostate cancer (RM-1) cells under hypoxic conditions (1% O_2_) showed a high cytotoxicity. Furthermore, the antitumor efficacy was evaluated using a cell-line-derived xenograft model by subcutaneously injecting RM-1 cells into the flank of C57BL/6 mice. Results showed that Dox-L prolonged the survival time in comparison with Dox-L without nitroimidazole derivate (34 vs. 25 days). In addition, researchers investigated the adverse effects of Dox-L on the body weights of the mice recorded during the treatment. Data revealed that Dox-L without nitroimidazole derivate showed less weight gain vs. Dox-L, indicating better tumor inhibition [141]. Recently, researchers have recognized that compared with nitroaromatics, azo has been the most promising hypoxia-responsive moiety for drug carriers. For example, Long et al. (2020) developed azo-based hypoxia-responsive hybrid L for Dox (Dox-HL) delivery, targeting tumor hypoxia. Dox-HL were studied under normoxic and hypoxic conditions. Results showed that Dox release in normoxic conditions was lower than under hypoxic conditions (20 vs. 80%). Also, hypoxia-triggered intratumoral drug release was assessed in tumor-bearing C57BL/6 and BALB/c nude mice, and the results demonstrated high hypoxia-triggered intratumoral drug release in the hypoxic tumor regions. In addition, in vivo antitumor efficacy was assessed and, as expected, Dox-HL had a high inhibition of tumor growth. The survival of mice was in good agreement with tumor growth inhibition, reaching 36 days [142].

As demonstrated above, azo-derivative molecules respond to the hypoxic regions and have been used as hypoxia-responsive nanocarriers in cancer. Additionally, azobenzene derivatives have a reduction potential that falls within the range of hypoxic environments [143]. Complex strategies and expensive materials to develop hypoxia-sensitive nanolipid formulations were used. Nano-drug delivery systems, such as cationic L, gained interest due to the tendency to reach the tumor microenvironment. However, cationic L are rapidly cleared by the MPS due to protein opsonization [144]. PEGylation can avoid negative effects in the clearance, creating a shielding layer at the surface of cationic L and, hence, the blood circulation [145]. So, Mashreghi et al. (2022) designed and synthesized a cationic liposomal Dox decorated with an azo-based linker (PEG-Azo-L) to enhance antitumor activity under hypoxic conditions. This activity was carried out using C26 tumor cells. In the hypoxic microenvironment, the azolinker cleavage was catalyzed by azobenzene reductase in hypoxia areas and exposed the positive charges of cationic L, indicating the efficacy of the PEG-Azo-L formulation [146]. In recent years, immunotherapy has been a major focus among tumor treatments, especially with metastatic cancers, where some patients, previously considered to be incurable, can survive [147]. The use of anti-PD-1 or anti-PD-L1 monoclonal antibodies exhibited satisfactory clinical responses in several cancers [148].

Recently, different studies confirmed the immunological effects of chemotherapy in combination with immune checkpoint inhibitor therapy [149]. In non-small-cell lung cancer, promising results were shown by combining chemotherapy of Pt with anti-PD-1/PD-L1 antibodies. Pt may upregulate PD-L1 expression in tumor tissue and exert a negative immunomodulatory effect, which can be counteracted by PD-1/PD-L1 inhibitors through their action pathway [150]. Also, some researchers recently confirmed that increased tumor oxygenation via different designed strategies could remarkably potentiate cancer treatments [151]. On approach, Song et al. (2022) studied metformin (met)-oxaliplatin (oxa)-loaded L (met-oxa-L) to potentiate cancer immunotherapy. Met-oxa-L was placed on CT26 tumors to evaluate the intratumoral hypoxia status. It was determined that the percentage of hypoxia-positive areas in tumor slices of mice was 3.84%. Furthermore, by monitoring the tumor suppression efficacy, met-oxa-L showed the highest tumor inhibition efficacy. In addition, these L were able to prime potent antitumor immune responses by inducing immunogenic cell death of cancer cells and promoting tumor oxygenation [152]. Chemotherapy can enhance antitumor responses through different mechanisms; however, some chemotherapy drugs would lead to upregulation of PD-L1 expression. Different from conventional chemotherapeutics causing PD-L1 enrichment, cyclopeptide RA-V (deoxybouvardin) catches our attention because of its satisfactory activities in cancer cells [153]. In view of this, Yao et al. (2022) designed a synergistic antitumor platform, BMS/RA@CC-L, using CT26 cancer-cell biomimetic nanoparticles, combining a chemotherapeutic drug (RA-V) and PD-1/PD-L1 against hypoxic tumor. These researchers incubated BMS/RA@CC-L with monocyte/macrophage-like cells to evaluate the antiphagocytosis capability, and this liposomal formulation exhibited remarkably decreased cellular uptake of BMS/RA@CC-L. Furthermore, to analyze the in vivo tumor-targeting ability, CT26 cell-membrane-coated L were injected into CT26 tumor-bearing nude mice. The mice treated exhibited a significant fluorescence enhancement in the tumor site compared to normal tissues (4 h vs. 24 h), indicating the high tumor specificity of BMS/RA@CC-L [154].

## 3. Dual/Multi-Responsive Liposomes

Stimuli-responsive L have emerged as a promising strategy in cancer therapy, leveraging the unique physiological and biochemical conditions of the tumor microenvironment to enable precise, controlled drug release. These nanocarriers are specifically designed to respond to endogenous signals, which are often dysregulated in tumor tissues. Additionally, they can be activated by exogenous stimuli, providing researchers with diverse mechanisms to trigger drug release [155,156]. This dual ability to harness both endogenous and exogenous clues makes stimuli-responsive L highly adaptable in overcoming challenges, such as inadequate drug targeting, accelerated clearance, and off-target toxicity.

**Figure 6:**
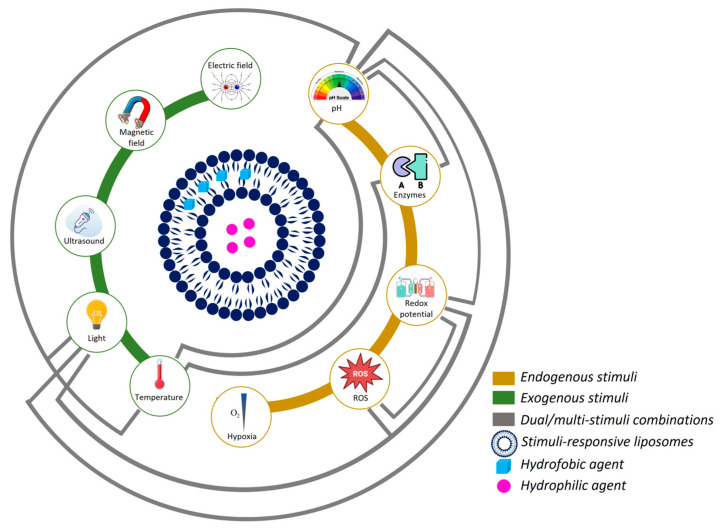
Diagram of dual/multi-stimuli-responsive liposomes (L): The gray lines represent specific combinations of stimuli that activate the drug release system, reflecting interactions described in the review text. This design illustrates how the L can simultaneously respond to endogenous (in yellow) and exogenous (in green) stimuli, optimizing therapeutic efficacy.

In recent years, the integration of multiple stimuli within a single delivery system gained significant attention due to its potential to enhance therapeutic precision and efficacy. By combining two or more stimuli, these systems offer more refined control over drug release, ensuring that therapeutic agents are activated specifically at the site of action. This not only maximizes the drug effectiveness but also reduces systemic side effects, a critical concern in conventional chemotherapy [4]. Figure 6 provides an overview of the various endogenous and exogenous stimuli combined in recent studies, illustrating the versatility of these dual/multi-stimuli-responsive systems. The ability to optimize drug delivery through dual/multi-stimuli-responsive L represents a major advancement toward personalized cancer treatments, where therapy can be customized to match individual tumor characteristics and disease progression.

Among these systems, dual-stimuli-responsive L, which respond to both temperature and enzymatic activity, exemplify how such platforms can improve therapeutic outcomes. Lyu et al. (2018) developed a novel hydrogel system where L serve as noncovalent crosslinkers and carriers for controlled release of molecular payloads. This system is formed by CHOL-modified DNA copolymers and L, creating a matrix that responds to temperature and enzymatic activity. Upon exposure to heat or the enzyme EcoR I, a gel-to-sol transformation occurs, releasing L loaded with hydrophobic (DiIC18(5)) and hydrophilic (calcein) compounds. In vitro studies confirmed the nanoplatform’s biocompatibility, along with its injectable and self-healing properties, positioning it as a potential candidate for biomedical applications [157]. Similarly, Palmese et al. (2020) designed temperature-sensitive L crosslinked within a PEG hydrogel for dual-stimuli-responsive drug delivery. These L, composed of 1,2-dipalmitoyl-sn-glycero-3-phosphocholine (DPPC) and 1,2-distearoyl-*sn*-glycero-3-phosphoethanolamine-N-[maleimide (polyethylene glycol)-2000 (Mal), exhibited a phase transition at 41 °C, allowing temperature-induced drug release. Crosslinked into a MMP-sensitive PEG-peptide hydrogel, the L released Dox in response to both hyperthermia (42 °C) and enzymatic activity. The system demonstrated sustained drug release, achieving up to 80% under hyperthermia conditions while exhibiting minimal cytotoxicity in fibroblast cultures [158].

Based on the principle of combining multiple physiological stimuli, integrating pH sensitivity with temperature responsiveness provides additional advantages for drug delivery. In tumor environments, where acidic pH is common, pH-sensitive L offer a complementary mechanism to further enhance drug release. The combined effect of heat and pH may promote L destabilization under acidic conditions and accelerate drug release at elevated temperatures. This dual approach has demonstrated promising results in optimizing therapeutic outcomes. For example, Zhou et al. (2019) developed Pt-acridine hybrid agents encapsulated in phosphatidylcholine (PC) and CHOL-based L. Under acidic conditions, these L released 91.5% of their drug load within 24 h, compared to just 35.6% under neutral conditions. In vitro studies demonstrated the higher cytotoxicity of the hybrid agent over Cis against lung cancer cells (NCI-H460), suggesting a promising strategy to overcome Cis resistance in non-small-cell lung cancer [159].

Following this approach, Sentoukas et al. (2021) engineered chimeric L, incorporating functional copolymers to deliver curcumin. With the use of dual stimuli, the system achieved controlled release while maintaining stability across different conditions. The encapsulation efficiency and long-term release of curcumin highlighted the potential to reduce off-target effects and enhance the treatment precision in cancer therapy [160]. In another study, Sugimoto et al. (2017) employed methacrylate-copolymer-based L for controlled drug delivery. The L exhibited a triggered release of the drug under acidic and elevated-temperature conditions. The release kinetics were studied by changing the temperature and pH, showing that the L released more drug at pH 5.0 and 42 °C than at physiological conditions (pH 7.4 and 37 °C). The antitumor efficacy was evaluated using HeLa cells, demonstrating enhanced cytotoxicity under dual-stimuli conditions compared to single stimuli. This work further emphasizes the importance of precision in drug release for targeted therapies, particularly in cervical cancer treatment [161].

Ta et al. (2010) advanced the field by designing L functionalized with polymeric copolymers for delivering Dox in targeting tumors. The team synthesized these copolymers using reversible addition-fragmentation chain transfer (RAFT) polymerization, incorporating N-isopropylacrylamide (NIPAAm) and propyl acrylic acid (PAA) to impart dual pH/temperature responsiveness. To assess the release properties, these polymer-modified L were tested for their membrane-disruptive behavior under modified pH and temperature conditions, showing enhanced drug release profiles. In addition, in serum stability studies, temperature-sensitive L demonstrated significantly lower drug leakage compared to traditional formulations, thus confirming its potential to reduce collateral damage to healthy tissues during cancer therapy [162].

**Figure 7:**
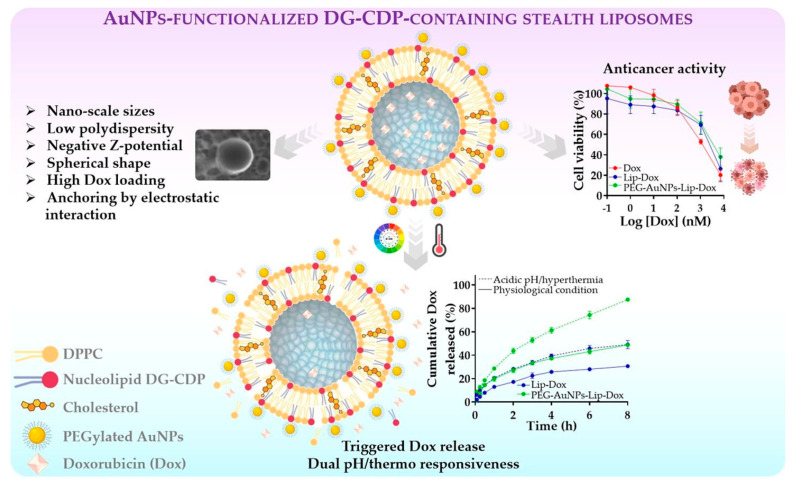
Representation of Dox-loaded nucleolipid-based liposomes (L) functionalized with PEGylated AuNPs, illustrating their dual sensitivity to pH and temperature changes for controlled Dox release in acidic environments and hyperthermia conditions. Highlighted properties include nanometric size, uniform size distribution, spherical structure, negative zeta potential, efficient drug loading, and stable anchoring of AuNPs via electrostatic interactions. These L exhibited increased therapeutic efficacy against breast and ovarian cancer cells. Dox: doxorubicin; AuNPs: gold nanoparticles; DPPC: dipalmitoylphosphatidylcholine; DG-CDP: 1,2-dipalmitoyl-sn-glycero-3-(cytidine diphosphate); Lip-Dox: nucleolipid-containing Dox-loaded liposomes; PEG-AuNPs-Lip-Dox: NH2-PEGylated AuNPs anchored on the surface of Lip-Dox. Reprinted with permission from Ref. [166].

Stealth L have been engineered to evade immune recognition and extend the circulation time, thereby enhancing drug accumulation at tumor sites. While PEGylation remains the most widely used strategy to achieve this effect [163], alternative approaches have also been explored. Zwitterionic lipids, such as DPPC, have been reported to confer stealth properties by reducing protein adsorption and minimizing recognition by the MPS. Similar to PEGylation, incorporating zwitterionic lipids into liposomal formulations has demonstrated an increase in the systemic circulation time [164]. Based on the use of zwitterionic lipids, García et al. (2021) investigated the impact of CHOL levels on the performance of thermosensitive L functionalized with gold nanoparticles (AuNPs) for controlled delivery of Dox. Two liposomal formulations, L1 (3.35 mol% CHOL) and L2 (40 mol% CHOL), were evaluated. The L were prepared using DPPC and didodecyldimethylammonium bromide (DDAB), and Dox was loaded using a transmembrane pH-gradient method. AuNPs were electrostatically anchored to the cationic liposomal surfaces. The study revealed that higher CHOL levels enhanced membrane rigidity, reduced L size, and increased drug encapsulation efficiency. In vitro Dox release studies demonstrated controlled drug release, with higher temperatures (42 °C) significantly enhancing Dox diffusion due to increased membrane permeability. Functionalization with AuNPs further improved thermal responsiveness and release profiles, particularly for the L2 formulation. Cytotoxicity assays in breast (MDA-MB-231) and ovarian (SK-OV-3) cancer cells confirmed the sustained antiproliferative activity of Dox-loaded L, with AuNPs-L2-Dox exhibiting enhanced thermosensitivity. These findings highlight the potential of CHOL-modulated, AuNP-functionalized L for hyperthermia-based cancer therapy [165]. Additionally, García et al. (2022) developed and evaluated an innovative approach using L composed of nucleolipids, DPPC, and CHOL, which were responsive to dual stimuli (temperature and pH changes). These L were functionalized with PEGylated-AuNPs to enable targeted delivery of Dox in cancer therapy. The Dox release profiles were studied under various pH and temperature conditions, revealing that the L remained stable at physiological pH and temperature but showed a significant increase in drug release under lower pH and higher temperature, confirming their dual sensitivity. Additionally, In vitro studies on cellular uptake and cytotoxicity in ovarian and breast cancer cells demonstrated enhanced anticancer efficacy of Dox-loaded L compared to free Dox [166], as shown in Figure 7.

Expanding on combination therapies, Nezhadali et al. (2020) developed L for the co-delivery of Dox and mitomycin C, aiming to enhance the efficacy of combination chemotherapy. The L were modified with a copolymer composed of NIPAAm for thermosensitivity and PAA for pH sensitivity. This formulation allowed controlled release under mildly acidic conditions (pH 5.5) and at elevated temperatures (42 °C), replicating tumor microenvironments. In vitro studies with breast cancer cells (MCF-7) demonstrated that the co-loaded L exhibited significantly higher cytotoxicity compared to free drug combinations, confirming the potential of the system to improve cancer treatment [167]. Furthermore, Zhao et al. (2020) developed L for the controlled release of cytarabine, a chemotherapeutic agent. The L were composed of DPPC and CHOL, providing thermosensitivity, while the exogenous layer was functionalized with poly (aspartic acid) grafted with octylamine for pH sensitivity. These dual-sensitive L were designed to increase release cytarabine in response to the acidic and hyperthermal conditions typically found in tumor environments (pH 5.0 and 42 °C). In vitro experiments using HepG2 liver cancer cells demonstrated that the L induced significantly higher cytotoxicity under these dual-stimuli conditions, compared to the free drug and single-stimuli L. Additionally, the system showed minimal toxicity to normal hepatocyte (L02) cells, indicating its potential for enhanced tumor targeting with reduced side effects [168].

In parallel, some L combine pH with enzymatic responsiveness, where enzymes overexpressed in the tumor microenvironment trigger structural changes in the carrier, leading to selective drug delivery. Ge et al. (2024) developed a tumor-specific liposomal nanoreactor system co-modified with human serum albumin and sialic acid, which was designed to enhance cooperative cancer therapy. These nanoreactors were activated in response to acidic pH and overexpressed enzymes within the tumor microenvironment, triggering a cascade of reactions to improve therapeutic selectivity. The researchers evaluated the biocompatibility of the serum albumin/sialic-acid-modified L In vitro, observing reduced phagocytosis by immune cells. In vivo studies, where the L were injected into tumor-bearing mice, demonstrated significant accumulation at the tumor site compared to normal tissues, indicating enhanced tumor targeting and improved therapeutic efficacy [169]. Also, Liu et al. (2019) developed dual-responsive polymer-L designed for synergistic cancer treatment via immuno-chemotherapy. These L respond to both the acidic pH of the tumor microenvironment and tumor-overexpressed enzymes, triggering the release of Dox in a controlled manner. The system enhances selective drug delivery and minimizes off-target effects. In vitro studies demonstrated improved cellular uptake and immune activation, while in vivo experiments in tumor-bearing mice showed significant tumor accumulation and a potent antitumor response. These results highlight the potential of this system for enhanced chemotherapy and immune modulation in cancer treatment [170].

Further advancements include systems that integrate pH/redox responsiveness, leveraging the acidic extracellular environment and intracellular redox imbalances. These carriers ensure that the release occurs only within specific cellular compartments, enhancing the therapeutic index. Zhang and Zhao (2011) investigated a strategy that utilized redox and pH stimuli to trigger drug release from L. They developed polymerized L composed of PEG, CHOL, and lipids containing S-S bonds, which could be cleaved in response to redox triggers. These L were loaded with Dox and tested for their responsiveness to redox and pH changes. In vitro experiments showed higher release under reducing conditions and at lower pH levels. Additionally, these L effectively targeted and eliminated cancer cells while minimizing effects on healthy cells. Recent advances in the development of dual pH/redox-responsive nanocarriers highlight the continuous progress in enhancing targeted drug delivery systems for inflammation treatment and cancer therapy [171]. Mavuso et al. (2016) focused on a dual nanoliposomal complex that incorporated copper-ligand coordination for chronic inflammation treatment. Their formulation utilized Eudragit^®^ E100-cystamine and phospholipids to enable the controlled release of a copper-liganded bioactive complex, specifically prednisolone succinate. This design allowed the L to release their therapeutic payload in response to the unique acidic and reductive conditions found in inflamed tissues. In vitro studies revealed that these copper-ligand L exhibited potent anti-inflammatory effects by regulating critical mechanisms involved in inflammation. Moreover, in vivo experiments using inflammation-induced animal models demonstrated a significant reduction in inflammatory markers and tissue damage when treated with this bioactive complex, underscoring its therapeutic potential in chronic inflammatory conditions [172]. Expanding on these developments, Li et al. (2019) introduced a pH/redox-responsive supramolecular structure (Pep-V⊂P-PEG) designed to enhance interactions with lipid membranes in cancer therapy. This system incorporated a peptide modified with viologen (Pep-V) and PEG-pillar[5]arene as a protective “helmet”. The architecture allowed the complex to modulate its conformation in response to the acidic and reductive conditions prevalent in tumor environments. They prepared L using neutral DPPC to represent normal cell membranes and negatively charged 1,2-dipalmitoyl-sn-glycero-3-phosphate (DPPG) to model tumor cell membranes. The results indicated that Pep-V demonstrated significant interactions with lipid membranes, enhancing stability at acidic pH while effectively destabilizing membranes upon exposure to reducing agents, like sodium thiosulfate (Na₂S₂O₃). Although this system did not carry a conventional chemotherapeutic agent, its design aimed to improve the delivery and efficacy of existing treatments through targeted membrane disruption [173].

**Figure 8:**
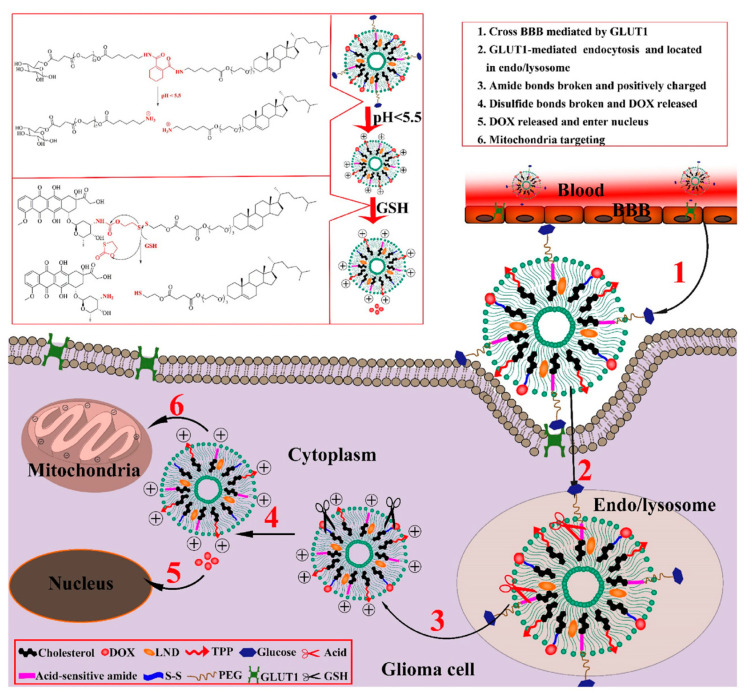
The synergistic anti-glioma process of doxorubicin (Dox) prodrugs and lonidamine (LND) co-loaded in pH-redox-responsive cascade-targeted liposomes modified with glucose and TPP (triphenylphosphonium). BBB: blood–brain barrier; GLUT1: glucose transporters; GSH: glutathione; S-S: disulfide bonds; PEG: polyethylene glycol. Reprinted with permission from Ref. [174].

More recently, Zhao et al. (2022) designed a pH/redox-sensitive cascade liposomal system using PEGylated glucose and TPP as targeting moieties for co-delivery of a prodrug of Dox and LND (Dox-LND@pH-redox-L) specifically targeting glioma. This innovative system was engineered to release its therapeutic cargo in the tumor microenvironment under acidic and reductive conditions, significantly improving drug activation and targeting specificity, as shown in Figure 8. The researchers conducted in vitro efficacy evaluations, demonstrating a marked increase in selective drug release and cytotoxicity in glioma cells. Furthermore, in vivo studies using glioma-bearing mice confirmed that the Dox-LND@pH-redox-L accumulated preferentially at the tumor site, resulting in significant tumor growth inhibition, and minimized off-target effects compared to non-targeted formulations. Together, these studies represent a significant progression in the field of responsive nanocarrier systems, highlighting the potential for improved treatment efficacy and targeting specificity in both cancer therapy and inflammation management [174].

Continuing along this gradient of stimuli, the interaction between redox and ROS-responsive L provides another level of control, such as oxidative stress within tumor cells, which can serve as a powerful trigger for drug release. Xue et al. (2022) designed Fe^3^⁺-mediated L co-loaded with shikonin and pyropheophorbide-a (PPA) to induce robust immunogenic cell death by integrating ROS enhancement and GSH depletion, responding to oxidative stress and redox imbalances in the tumor microenvironment. These researchers incubated the Fe^3^⁺/shikonin/PPA-loaded L with 4T1 breast cancer cells to evaluate their intracellular ROS production, showing a significant ROS increase while simultaneously reducing GSH. Furthermore, to analyze the in vivo antitumor efficacy, these L were injected into 4T1 tumor-bearing mice, and the results showed significant tumor growth inhibition. Treated mice exhibited enhanced immunogenic cell death markers, such as calreticulin exposure and ATP release, highlighting the potent antitumor immune response triggered by this dual-stimuli liposomal system [175].

Similarly, ROS/glucose-responsive L take advantage of the metabolic alterations in tumors, where elevated glucose uptake and oxidative stress act as dual triggers for controlled release. Zhang et al. (2018) developed glucose/oxygen-exhausting L for combined cancer metabolism inhibition and hypoxia-activated therapy. These L encapsulated GOx and the hypoxia-activated prodrug AQ4N (banoxantrone dihydrochloride), formulated from DPPC, CHOL, and DSPE-mPEG5k (PEG5k-conjugated distearoilphosphatidylethanolamine), achieving an average size of approximately 100 nm. The L-GOx effectively catalyzed the conversion of glucose and oxygen into gluconic acid and H₂O₂, increasing oxidative stress in cancer cells. In vivo studies in 4T1 tumor-bearing mice demonstrated that L-GOx and L-AQ4N exhibited prolonged blood circulation and significant tumor accumulation. Notably, L-GOx reduced intratumoral oxygen levels, enhancing hypoxia markers. The combination of these therapies led to superior tumor growth inhibition, underscoring the potential of this dual approach for effective cancer treatment [176].

The versatility of these systems becomes even more apparent when multiple types of stimuli are integrated. While most reported platforms respond to two endogenous triggers, the combination of endogenous and exogenous stimuli offers additional layers of control. These multi-stimuli systems combine endogenous cues, such as pH, temperature, or redox potential, with exogenous triggers like light or magnetic fields, further enhancing drug release precision. Yao et al. (2021) explored the development of hybrid L that are triple-responsive and designed for targeted tumor diagnosis and treatment, with strong magnetic resonance imaging capabilities. They created both anisamide-modified and non-modified molecules responsive to UV light and GSH, as well as celecoxib-modified and non-modified molecules responsive to UV light and H_2_O_2_. The final formulation of these UV/GSH/H_2_O_2_ triple-responsive L was achieved by combining the molecules (10,10-NB-S-S-P-AA and 10,10-NB-OA-P-CE) with a contrast agent (12,12-NB-DTPA-Gd). The resulting system displayed excellent drug encapsulation efficiency, along with good stability and biocompatibility, while also delivering outstanding magnetic resonance imaging performance. Under conditions simulating low pH, H_2_O_2_, and GSH, the L released up to 80% of the encapsulated drug. Moreover, the drug-loaded L exhibited effective targeting of tumor cells. Overall, this triple-responsive L system shows potential as a promising approach for tumor theranostics [177].

Alternatively, Torres et al. (2025) investigated the improvement of AuNPs’ functionalization on Dox-loaded L using quality-by-design tools. They explored a range of variables that influence the properties of the final system, AuNPs-L-Dox, and established optimized conditions to improve the interfacial characteristics and maximize Dox encapsulation efficiency. In vitro experiments demonstrated that Dox release was effectively controlled by pH variations and heat generated from light exposure, with a notable increase in the release under acidic conditions and during optical hyperthermia. The AuNPs-L-Dox showed significant antitumor activity against the SK-OV-3 ovarian cancer cell line, demonstrating a response that was both dependent on the drug concentration and influenced by irradiation-induced temperature changes. Safety evaluations in HaCaT keratinocyte cells indicated that the biocompatibility of AuNPs-L-Dox was comparable to that of free Dox [178].

Similarly, trackable L have emerged as promising dual-function systems, integrating diagnostic and therapeutic capabilities to advance personalized treatments. For instance, Li et al. (2024) synthetized pH/oxygen/photothermal-sensitive L to diagnose solid tumors in the early stage. These L were synthesized using a pH-sensitive amphiphilic ruthenium complex, which acts as an oxygen-sensitive fluorescent probe, co-assembled with lipids to encapsulate indocyanine green (ICG) as a photothermal agent. The L exhibited enhanced tumor detection through oxygen concentration monitoring and radiometric imaging, which distinguished tumor sites based on hypoxia. Under near-infrared (NIR) light, the L facilitated precise photothermal therapy, achieving efficient tumor cell apoptosis In vitro and significant tumor growth inhibition in vivo. The pH sensitivity also promoted higher liposomal uptake in tumor tissues, improving both diagnostic imaging and therapeutic efficacy. This dual-function system demonstrates the potential for clinical translation, combining diagnostic and therapeutic capabilities to enhance cancer treatment outcomes [179].

Tasnim et al. (2024) developed NIR-light-responsive L co-loaded with Ptx and ICG for simultaneous tracking and photothermally triggered drug release. These L were designed to improve precision in cancer therapy, particularly for skin tumors. In vivo tracking using a NIR imaging system demonstrated efficient Ptx release in acidic environments and under mild hyperthermia [180]. Additionally, Chen et al. (2017) designed luminescence-trackable L (L-PLNPs) by integrating persistent luminescence nanoparticles (PLNPs) with L for real-time imaging and chemotherapy. The system used long-lasting, NIR-emitting luminescence from PLNPs, which could be reactivated by red LED light, achieving high Ptx loading efficiency. In vitro studies confirmed efficient internalization into MCF-7 cells, significantly reducing cell viability. In vivo, L-PLNPs passively accumulated in tumors via the EPR effect, allowing precise tracking and effective tumor inhibition in mouse models [181]. Similarly, Payne et al. (2024) developed and evaluated dual-labeled L for enhanced drug delivery monitoring in brain therapies using focused ultrasound (FUS)-mediated BBB opening. These L incorporated gadolinium for magnetic resonance imaging (MRI) and NIR fluorescent dye (CW800) for bimodal detection. In vivo, 9.4-T MRI confirmed L delivery, while ex vivo NIR fluorescence imaging validated their accumulation in mouse models. Passive cavitation detection revealed that acoustic signals correlated with MRI contrast, enabling real-time tracking of BBB opening and L delivery. The study demonstrated that 110 nm of L effectively crossed the BBB, with their spatiotemporal distribution monitored up to 4 h post-treatment. This innovative approach provided real-time, quantitative feedback on drug delivery, highlighting its potential to optimize brain tumor therapies while maintaining safety [182]. Another advancement was reported by Moloney et al. (2023), who designed solid magnetoliposomes (SMLs) as MRI-trackable. These SMLs consisted of solid magnetic cores encapsulated within a phospholipid bilayer, with a tunable outer monolayer incorporating functionalities, such as PEG. SMLs demonstrated advanced non-invasive MRI tracking capabilities, enabling precise detection of tumor deposition in patient-derived pancreatic adenocarcinoma models. The study showed that SMLs exhibited prolonged stability, sustained tumor accumulation, and enhanced sensitivity for detecting tumor microenvironment changes induced by stromal modulation strategies, such as sonic hedgehog pathway inhibitors [183].

Although the focus of this review is on L responsive to endogenous stimuli, it is essential to mention these multi-stimuli systems due to their growing importance and therapeutic potential. These hybrid approaches, combining endogenous and exogenous stimuli, exemplify the advancements in precision medicine aimed at overcoming the limitations of conventional therapies. The continuous development of multi-responsive liposomal systems demonstrates the potential of these carriers to address the challenges associated with traditional drug delivery. While preclinical studies have shown promising results, further work is required to refine these systems and ensure their successful translation into clinical applications. Stimuli-responsive L, with their ability to integrate multiple triggers, represent a significant advancement in achieving more precise and effective cancer therapies.

## 4. Remarks and Future Perspectives

Advances in the encapsulation of antitumor drugs within nanocarriers, particularly stimuli-responsive L, proved to be a promising strategy for enhancing the safety and efficacy of cancer treatments. This review emphasized the variety of stimuli-responsive L that can detect and respond to endogenous signals, allowing for controlled release of the encapsulated drug. These stimuli cover a diverse array, including pH changes, ROS levels, enzymatic activity, redox potential, and temperature changes, among others. This specific responsiveness not only enhances precision in drug delivery but also minimizes adverse effects on healthy tissues, overcoming some of the limitations of conventional drug delivery systems.

Even through their potential to enhance cancer treatments, the clinical application of stimuli-responsive L still faces substantial obstacles. To ensure they can be safely and effectively used in patients, it is crucial to conduct detailed studies on their pharmacokinetics, distribution within the body, and long-term safety. Additionally, extensive testing in advanced animal models, followed by human trials, will be needed to establish their reliability and therapeutic impact in clinical environments. These studies will not only provide a more detailed understanding of the interactions between these nanocarriers and living organisms but also facilitate the validation of their potential clinical applications.

Multi-responsive systems that combine multiple activation signals have demonstrated superior control over drug release, offering a pathway to overcoming common barriers, such as rapid clearance and off-target toxicity. These characteristics are particularly promising for the treatment of neoplasms resistant to conventional chemotherapy, as they allow for both temporal and spatial modulation of drug release. However, to bring these systems closer to clinical practice, it remains necessary to optimize aspects such as their stability in circulation, scalability of manufacturing, and long-term safety profiles.

To address these challenges, adopting multidisciplinary approaches that integrate advances in materials science, pharmaceutical engineering, and regulatory frameworks is essential. Such collaborative efforts will help bridge the gap between laboratory research and clinical implementation, particularly by tackling technological and logistical hurdles. For example, the manufacturing processes for these advanced systems often rely on sophisticated and resource-intensive techniques, which can hinder their scalability. Developing standardized, cost-effective protocols that ensure batch-to-batch consistency and adherence to good manufacturing practices will be critical for facilitating industrial-scale production without compromising product quality and reproducibility.

From a regulatory perspective, the hybrid nature of these multifunctional nanocarriers adds layers of complexity to their approval pathways. These systems often integrate both diagnostic and therapeutic functionalities within a single platform, requiring extensive documentation and rigorous testing to demonstrate their safety and efficacy. Regulatory agencies, such as the FDA and EMA, mandate clear evidence for preclinical evaluations and clinical trial outcomes. This underscores the need for close collaboration between researchers, industry professionals, and regulatory bodies to establish transparent guidelines for their development and approval.

Looking ahead, integrating molecular docking studies could optimize the specific localization and release of drugs at tumor sites. Additionally, incorporating advanced knowledge of the tumor microenvironment will enable the development of safer, more effective, and economical nanomedicines that respond precisely to the biological conditions of different cancer types. With these efforts, stimuli-responsive L are emerging as a revolutionary technology in precision medicine, with the potential to redefine antitumor therapies and significantly improve clinical outcomes in oncology.

## Figures and Tables

**Table 1:** Examples of pH-responsive liposomes, including their main characteristics and stage of development.

Liposome Composition	Therapeutic Agents Loaded	Biomedical Application	Stage of Development	Ref.
HSPC, CHOL, and GC	Dox	Fibrosarcoma	Preclinical (murine xenograft tumor model)	[25]
MGlu, HA, and CHex-HA	Dox	Cervix cancer Breast cancer	Preclinical (murine colon model)	[26]
CHOL, DSPE, PEG_2000_, and SPC	Ptx	Melanoma Breast cancer	Preclinical (tumor-bearing mice)	[27]
Peptide KLA, DMA, DSPE, and SPC	Ptx	Lung cancer	Preclinical (nude mice)	[28]
EYPC and MGlu-HPG	Ovalbumin	Lymphoma	Preclinical (C57BL/6 mice)	[29]
DMAP, SPC, PEG, DSPE, and DCC	Dox	Melanoma	In vitro (A375 cancer cell line)	[30]
EYPC, DOPE, MGlu, MPLA, and DEX	Ovalbumin	Lymphoma	Preclinical (C57BL/6 mice)	[31]
DPPC, CHOL, and DC	Dox	Osteosarcoma	In vitro (K7M2 and NIH/3T3 cancer cell lines)	[32]
PC, DOPE, DSPE, PEG_2000_, and HA	Dox	Breast cancer	In vitro (MCF-7 cancer cell line)	[33]
HSPC, DOPE, DSPE, PEG_2000_, and CHOL	Dox	Breast cancer	Preclinical (BALB/c mice)	[34]
DSPE, PEG_2000_, and CHOL	Emtansine	Breast cancer	Preclinical (murine RAW 264.7)	[35]
CHEMS, DOPE, DSPE, PEG_2000_, and CHOL	Gemcitabine	Pancreatic cancer	Preclinical (MIA PaCa-2 cells)	[36]
EPC, CHEMS, DOPE, DSPE, PEG_2000_, and CHOL	Dox	Glioma	Preclinical (BALB/c nude mice)	[37]
CHEMS, DOPE, SPE, and PEG_2000_	5-Fluorouracil	Breast cancer	In vitro (MDA-MB-231 and SK-BR-3 cancer cell lines)	[38]
CHOL, SPC, DSPE, and PEG_5000_	Bovine serum albumin	Bladder cancer	Preclinical (C57BL/6 mice)	[39]
DOPE, CHOL, and DC	Resiquimod	Colorectal cancer	Preclinical (BALB/c mice)	[40]
CHEMS, DOPE, DSPE, and PEG_2000_	Radiotracer (99mTc-HYNIC-βAla-Bombesin(7–14))	Breast cancer	Preclinical (BALB/c mice)	[41]
CHEMS, DPPC, DSPE, and PEG_2000_	Docetaxel Doxycycline hyclate	Lung cancer	Preclinical (BALB/c mice)	[42]
HSPC, HA, and DEAP	Docetaxel	Colon carcinoma	Preclinical (BALB/c mice)	[43]
DOPE, DOTAP, and PC	Docetaxel	Breast cancer	Preclinical (Swiss albino mice)	[44]
DOPE, CHEMS, DSPE, and PEG_750_ or PEG_2000_	Calcein	Glioblastoma	Preclinical (murine model of glioblastoma)	[45]
SPC and CHOL	Paclitaxel hydroxypropyl-β-cyclodextrin complex	Lung cancer	Preclinical (BALB/c mice)	[46]
DOPE, CHEMS, DSPE, CHOL, HSPC, and PEG_2000_	Dox	Cervical cancer	In vitro (HeLa cancer cell line)	[24]
DOPE, CHEMS, DSPE, and PEG_2000_	Dox	Breast cancer	Preclinical (BALB/c mice)	[47]
DOPE, CHEMS, CHOL, DSPE, and PEG_2000_	Peptidomimetic-doxorubicin conjugate	Lung cancer Breast cancer	Preclinical (BALB/c nude mice)	[48]
DOPE, CHOL, DSPE, PEG_2000_, and CL	Daunorubicin	Melanoma	In vitro (B16-BL6 cancer cell line)	[49]
DOPE, CHEMS, DSPE, and PEG_2000_	Simvastatin Dox	Breast cancer	In vitro (MDA-MB-231, MCF-7, and SK-BR-3 cancer cell lines)	[50]
DOPE, CHEMS, DSPE, PEG_2000_, and EDC	Docetaxel	Breast cancer	Preclinical (Dawley rats)	[51]
PC, CHOL, and CHEMS	Morin	Hepatocellular cancer Breast cancer Lung cancer Gastric cancer	Preclinical (BALB/c nude mice)	[52]
DPPC, DSPE, PEG_2000_, DOPE, and CHOL	Echinomycin	Breast cancer Lung cancer	In vitro (MDA-MB-231, MCF-7, and A549 cancer cell lines)	[53]
DOPE, CHEMS, and PEG_2000_	Cu(II) complex (Cu(1,10- phenanthroline)Cl2)	Colorectal carcinoma	Preclinical (BALB/c mice)	[54]
DOPE, CHEMS, CHOL, DSPE, and PEG_2000_	Irinotecan	Colorectal carcinoma	Preclinical (BALB/c mice)	[55]
DPPE and PEG_2000_	Imidazole	Cervical cancer Breast cancer Lung cancer	In vitro (MDA-MB-231, MDA-MB-468, and A549 cancer cell lines)	[56]
DOTAP, DOPE, and CHOL	Sorafenib and VEGF-siRN	Hepatocellular carcinoma	Preclinical (Kunming mice)	[57]
Eudragit^®^ S100, OA, CHOL, and SC	Curcumin	Colon cancer	In vitro (Caco-2 cancer cell line)	[58]
EPC and CHEMS	Carboplatin	Lung cancer	In vitro (A549 cancer cell line)	[59]

Abbreviations: HSPC: hydrogenated soy phosphatidylcholine; CHOL: cholesterol; GC: glycol chitosan; MGlu: 3-methylglutarylated; HA: hyaluronic acid; CHex-HA: 2-carboxy cyclohexane-1-carboxylate; DSPE: 1,2-distearoyl-sn-glycero-3-phosphoethanolamine; PEG: poly(ethylene glycol); SPC: soybean phospholipids; KLA: peptide; DMA: dimethylmaleic anhydride; EYPC: egg yolk phosphatidylcholine; MGlu-HPG: 3-methylglutarylated hyperbranched; DMAP: dimethylamino pyridine; DCC: dicyclohexylcarbodiimide; DOPE: L-dioleoyl phosphatidylethanolamine; MPLA: monophosphoryl lipid A; DEX: dextran; DPPC: 1,2-dipalmitoyl-sn-glycero-3-phosphocholine; DC: dimethylaminoethane-carbamoyl; EPC: egg phosphatidylcholine; CHEMS: cholesteryl hemisuccinate; DEAP: 3-diethylaminopropyl; PC: phosphatidylcholine; DOTAP: 1,2-dioleoyl-3-trimethylammonium propane; CL: cardiolipin; EDC: ethyl carbodiimide hydrochloride; DSPC: 1,2-distearoyl-sn-glycero-3-phosphocholine; DPPE: 1,2-dipalmitoyl-sn-glycero-3-phosphoethanolamine; OA: oleic acid; SC: sodium cholate; ES100: Eudragit^®^ S 100.
